# Changing profiles of the burden of Alzheimer's disease and other dementias attributable to smoking in the belt and road initiative countries: A secondary analysis of global burden of disease 2019

**DOI:** 10.1016/j.heliyon.2024.e27935

**Published:** 2024-03-13

**Authors:** Zheng Luo, Xin He, Huihui Lv, Qizhe Wang, Wenchang Jia, Yajun Zhao, Xinyi Li, Jiali Yu, Hongyu Hao, Yun Bao, Nuo Chen, Xiaopan Li

**Affiliations:** a Department of Neurology, Shanghai University of Medicine & Health Sciences Affiliated Zhoupu Hospital, Shanghai, 200316, China; b Department of Neurology, Kaifeng 155 Hospital, Henan, 475003, China; c Department of Neurology, Yueyang Hospital of Integrated Traditional Chinese and Western Medicine, Shanghai University of Traditional Chinese Medicine, Shanghai, 200437, China; d Department of Health Management Center, Zhongshan Hospital, Shanghai Medical College of Fudan University, Shanghai, 200032, China; e School of Public Health, Fudan University, Shanghai, 200032, China

**Keywords:** Belt and Road initiative countries, Dementia, Burden of disease, Smoking, Disability-adjusted life years, Trend analysis

## Abstract

**Objectives:**

This study was aimed at analyzing the burden and trend of Alzheimer's disease and other dementias attributed to smoking (SADD) in the Belt and Road Initiative (BRI) countries during 1990–2019.

**Methods:**

Data from The 2019 Global Burden of Disease Study was used to extract information on the burden of SADD in terms of the numbers and age-standardized rate of mortality (ASMR) and disability-adjusted life years (ASDALR) in the BRI countries for 1990–2019. The average annual percent change (AAPC) was used to analyze the temporal trends of ASDALR from 1990 to 2019 and in the final decade by Joinpoint regression analysis.

**Results:**

The DALYs of SADD were the highest in China, India, and the Russian Federation in 1990 and in Lebanon, Montenegro and Bosnia, and Herzegovina in 2019. From 1990 to 2019, the ASDALR in China had increased from 55.50/10^5^ to 66.18/10^5^, but decreased from 2010 to 2019, while that of India had declined from 32.84/10^5^ to 29.35/10^5^, but increased from 2010 to 2019. The ASDALR showed the fastest increase in the Russian Federation, with AAPC of 1.97% (95% confidence interval [CI]: 1.77%, 2.16%), and the fastest decline in Sri Lanka, with AAPC of −2.69% (95% CI: 2.79%, −2.59%). ASMR and ASDALR from SADD showed a substantial decline during 1990–2019 both globally and in the different socio-demographic index (SDI) regions (all *P* < 0.05, except for the high-middle-SDI region). Compared to the rates in males, the AAPC in ASDALR of females was significantly greater in 20 countries(all *P* < 0.05). In the age group of 20–54 years, the DALYs rate showed a decreasing trend only in 13 members in the low-SDI region (all *P* < 0.05).

**Conclusion:**

Under the premise of eliminating the differences, mobilizing resources in the country itself, the BRI organization, and globally will help reduce the global SADD burden and achieve healthy and sustainable development.

## Abbreviations

ADD: Alzheimer's disease and other dementias
SADD: Alzheimer's disease and other dementias attributable to smoking
GBD: Global Burden of Disease
BRI: the Belt and Road Initiative
SDI: social development index
ASRs: age-standardized rates
YLDs: years lived with disability
YLLs: years of life lost
DALYs: disability-adjusted life years
ASDALR: age-standardized disability-adjusted life years rate
ASMR: age-standardized mortality rate
AAPC: average annual percent change
UI: uncertainty interval
CI: confidence interval
IHME: Institute for Health Metrics and Evaluation
WHO: World Health Organization

## 1 Introduction

Alzheimer's disease and other dementias (ADD) are major global public health issues for which there are no specific prevention or treatment measures at present [1]. Over the next 30 years, the number of dementia patients worldwide is estimated to increase from 50 million to 152 million. ADD is the fifth leading cause of death globally and the second leading cause of neurological death [1]. The prevalence of ADD varies greatly between different regions and countries, while the incidence is higher in low-income countries than in high-income countries; additionally, the burden of these diseases increases with aging [2]. Studies have been previously conducted on the changing trends of ADD globally; the distribution of dementia based on gender, age, country, and region; as well as the risks of developing dementia [1,[3], [4], [5]]. However, research is limited on dementia prevention and burden reduction in the context of close cooperation between international political and economic organizations. Studies have also identified various risk factors for dementia, including but not limited to aging, hypertension, obesity, diabetes, physical inactivity, hearing loss, smoking, depression, low education levels, excessive alcohol consumption, brain injury, air pollution, and low frequency of social contact; furthermore, quitting smoking in later life has been reported as the best measure for preventing dementia and reducing the number of dementia patients by 5% [6]. The individual-level tobacco control policies put forth by the World Health Organization (WHO) have been widely accepted by countries, but the degree of their implementation varies. In this light, there is potential for mutual international communication and collaboration [[7], [8], [9], [10], [11]]. To effectively achieve the goal of rapidly eliminating tobacco-related deaths and diseases, some researchers highlighted the need for holding another strategic dialogue on harm reduction, preferably led by a government agency, a scientific organization, and/or respected scientists who are not closely associated with a specific ideology [11]. Consequently, the Belt and Road Initiative (BRI) was established between countries that were closely associated via long-term trade; together, these countries account for over 60% of the world's population [12]. Consumption of tobacco products has been found to be a leading risk factor for cancer in the BRI countries [13]. The adoption of China's tobacco control policy, 2015, appears to have reduced the number of hospitalizations for all cardiovascular diseases by more than 10%, while increasing the number of hospitalizations due to cerebrovascular diseases by over 20% [14]. China National Tobacco Corporation, the world's largest cigarette manufacturer, has also released a "Tobacco Industry in Belt and Road Development Plan" [8,15].

Under these circumstances, analyzing the trends in the burden of ADD attributed to smoking in the BRI countries can help understand the differences between the countries and explore solutions to the structural imbalances and inequalities in tobacco control policies. This can, in turn, help develop strategies for the prevention or delay of dementia and achieve sustainable development goals by reducing non-communicable disease mortality in the BRI countries [16] and improving people's health by strengthening multilateral cooperation and complementing each other's strengths.

## 2 Methods

### 2.1 Data sources

In this study, data for the 66 BRI countries, as described elsewhere, were included [17]. The 2019 Global Burden of Disease Study, which is an international collaborative surveillance system, includes data on 369 diseases and injuries, 87 risk factors, and combinations of risk factors across 204 countries and territories from 1990 to 2019. The detailed methodology of the data handling has been published elsewhere [[18], [19], [20]]. DALYs is a composite indicator of the burden of disability and premature death due to disease and is obtained as a total of YLLs (number of deaths x standard life expectancy at age of death) and YLDs [20]. In this study, data on the annual smoking-induced Alzheimer's disease and other dementias (SADD) deaths, DALYs, and respective age-standardized rate (ASR) by gender and age from 1990 to 2019 in BRI countries was extracted from the Institute for Health Metrics and Evaluation website (https://vizhub.healthdata.org/gbd-results/). To ensure replicability and transparency of results, our study was conducted in accordance with the Guidelines for Accurate and Transparent Health Estimates Reporting (GATHER) (Table S1) [21].

### 2.2 Statistical analyses

The absolute number of cases, mortality, and DALYs for SADD in BRI countries were determined. For estimated metrics, the 95% uncertainty interval (UI) were calculated by drawing 1000 times from each number of posterior distributions, using the 2.5th and 97.5th orders of the uncertainty distribution [18]. ASR (Σ(weighted age-specific rates) for mortality and DALYs were estimated using a global age structure from 2019, which allow comparisons across time, countries, and subregions. We defined four age groups: 20–54 years, 55–59 years, 60–79 years, and ≥80 years. Data were stratified by regions defined in terms of socio-demographic index (SDI): high-, high-middle, middle-, low-middle, and low- SDI. The methods of SDI development and computation are detailed elsewhere [22]. The temporal trends of disease burden were assessed in terms of average annual percent change (AAPC) from 1990 to 2019 by using the Joinpoint regression software, and 95% confidence interval (CI) for the trend segment was identified. In addition, we evaluated AAPCs of age-standardized DALYs rate (ASDALR) for SADD, stratified by sex and age. Additionally, we compared the changes in the AAPC of burden in SADD during the last decade (2010–2019) and throughout the study period (1990–2019). All analyses were conducted using the Joinpoint Regression Program (Version 4.9.0.0. March 2021) [17]. Visualization of the maps of the BRI countries was performed using the “ggmap” package in R software (version 4.3.0, R core team). The “ggmap” package is an extension package that obtains shapefiles from Google Maps. *P* < 0.05 was considered statistically significant.

## 3 Results

The mortality and DALYs of SADD in BRI countries for 1990 and 2019 are shown in Table 1. An increase in the DALYs of SADD from 1990 to 2019 was noted both globally and in different SDI regions. In China the number of deaths and DALYs from SADD were 55530.52 (95% UI: 11948.61 to 151958.54) and 83249.31 (95% UI: 15628.64 to 151956.31) in 2019, indicating an increase of 0.83 million from the value in 1990 and accounting for 41.12% of the global increase in DALYs growth.

**Table 1:** The absolute number of mortality and DALYs for SADD in the “B&R” countries in 1990 and 2019.

	Mortality in 1990		Mortality in 2019		DALYs in 1990		DALYs in 2019	
Countries	Number	95%UI	Number	95%UI	Number	95%UI	Number	95%UI
**Global**	97823.72	(21915.73,274801.89)	214247.31	(47319.74,589537.48)	1945222.36	(772072.70,4486577.52)	3960865.24	(1593346.27,8903496.15)
**SDI Regions**								
Low SDI	2035.45	(408.96,6014.86)	4898.28	(1044.17,14128.57)	43166.00	(15563.09,106887.76)	93779.00	(33265.66,233004.92)
Low-middle SDI	8792.42	(1913.83,25259.18)	22443.23	(4833.78,63441.21)	181836.14	(67603.81,440364.23)	421476.13	(157699.85,1022123.01)
Middle SDI	18634.88	(4041.4,52910.71)	51757.35	(11589.4,147145.67)	393885.65	(142939.06,953280.38)	1048781.46	(410920.7,2447720.29)
High-middle SDI	23421.55	(5175.29,66643.06)	58787.31	(13048.68,164156.43)	496126.14	(196119.13,1163392.43)	1143690.54	(462973.78,2555317.83)
High SDI	44884.75	(10107.73,124271.99)	76252.01	(17618.26,207579.71)	829176.53	(346926.83,1876571.32)	1251228.56	(513875.93,2777214.46)
**East Asia**								
China	15781.94	(3350.27,46686.13)	55530.52	(11948.61,151958.54)	357257.80	(127874.64,866496.54)	1186180.56	(483817.45,2652571.50)
**Central Asia**								
Armenia	53.77	(12.07,151.56)	114.70	(25.71,317.40)	1045.55	(423.8,2377.33)	2025.98	(792.16,4678.56)
Azerbaijan	95.08	(21.23,262.85)	151.73	(33.17,434.98)	1677.09	(662.39,3887.38)	3200.39	(1291.62,7301.71)
Georgia	92.85	(19.69,267.55)	173.17	(37.88,482.91)	2028.53	(827.81,4670.77)	2942.08	(1174.08,6725.20)
Kazakhstan	156.46	(34.06,449.76)	197.16	(41.46,580.01)	3317.19	(1356.88,7825.13)	4442.18	(1753.58,10644.83)
Kyrgyzstan	62.58	(13.85,173.01)	89.41	(20.33,259.76)	1160.44	(475.43,2677.68)	1692.14	(673.97,4084.98)
Mongolia	17.32	(3.52,50.98)	32.11	(6.58,92.04)	366.31	(141.21,876.99)	710.55	(268.52,1672.75)
Tajikistan	84.41	(19.89,233.07)	45.96	(9.61,134.46)	1366.52	(544.17,3162.96)	1084.44	(410.35,2557.07)
Turkmenistan	34.92	(7.78,99.05)	47.21	(10.24,133.76)	699.19	(278.08,1618.80)	1003.78	(386.32,2367.76)
Uzbekistan	131.65	(28.82,390.79)	104.41	(22.18,310.24)	2457.78	(924.01,5824.53)	3002.77	(1150.60,7276.22)
**South Asia**								
Bangladesh	877.69	(190.27,2518.49)	2016.79	(422.54,5840.50)	17326.34	(6657.23,40751.26)	38753.62	(14588.33,92224.37)
Bhutan	1.78	(0.37,5.26)	6.95	(1.41,20.01)	38.90	(13.35,95.46)	118.38	(38.64,294.53)
India	3612.06	(743.19,10828.38)	14266.89	(2992.29,40609.24)	81077.65	(29401.41,207199.88)	250897.95	(87845.74,626899.39)
Nepal	143.99	(30.45,426.45)	453.02	(95.31,1323.47)	2953.73	(1062.25,7188.79)	8114.76	(2815.22,19999.16)
Pakistan	1285.59	(266.42,3748.28)	1427.77	(289.45,4290.24)	23532.17	(8744.16,58679.84)	28286.12	(10178.13,72097.48)
**Southeast Asia**								
Cambodia	99.56	(22.14,292.75)	330.27	(76.03,931.74)	2049.86	(747.09,5301.54)	6151.01	(2178.20,15053.46)
Indonesia	1198.90	(249.96,3484.93)	3258.80	(697.58,9629.76)	25748.80	(9618.86,63869.83)	66621.22	(23512.28,165314.79)
Laos	36.98	(8.08,104.94)	78.26	(17.00,233.83)	850.94	(325.76,2043.88)	1567.83	(552.22,3979.72)
Malaysia	215.11	(47.03,614.20)	484.97	(105.81,1422.30)	3949.76	(1422.50,9662.57)	9354.04	(3280.34,23006.35)
Maldives	1.84	(0.39,5.39)	8.12	(1.88,22.84)	40.81	(14.82,102.55)	140.73	(51.96,329.50)
Burma	641.72	(134.95,1827.93)	888.04	(171.96,2634.47)	13288.06	(4883.49,32160.11)	16427.45	(5467.58,40870.26)
Philippines	819.96	(175.23,2292.43)	1629.77	(343.83,4716)	16434.50	(5565.02,41107.04)	31798.15	(10982.24,77734.43)
Sri Lanka	196.57	(43.04,570.49)	262.80	(48.86,810.75)	3807.12	(1295.80,9431.59)	5067.12	(1585.65,12969.79)
Thailand	935.31	(207.97,2645.68)	2308.36	(502.95,6745.58)	18095.89	(6379.77,44769.49)	40124.21	(14319.94,98053.36)
Viet Nam	846.09	(188.84,2449.83)	2108.22	(454.76,5838.56)	16155.49	(5697.56,40555.09)	39113.25	(13602.91,94214.48)
**High-income Asia pacific**								
Brunei Darussalam	1.59	(0.33,4.56)	3.38	(0.71,9.99)	38.00	(14.26,91.92)	80.50	(28.96,196.77)
Singapore	38.23	(7.77,106.96)	109.28	(23.43,307.23)	767.82	(280.77,1856.71)	2120.32	(807.20,4907.21)
**North Africa and Middle East**								
Afghanistan	85.60	(16.95,248.87)	164.41	(33.81,495.91)	1644.56	(541.23,4190.48)	3113.82	(1044.92,7744.13)
Bahrain	3.26	(0.68,9.38)	12.88	(2.66,36.03)	78.42	(28.71,190.72)	327.70	(124.77,776.49)
Egypt	513.18	(109.95,1516.12)	1457.75	(320.81,4137.86)	10651.32	(4198.86,25354.49)	28805.65	(11546.59,66406.25)
Iran	397.03	(82.38,1128.68)	1466.17	(327.21,4079.79)	9459.03	(3526.05,22462.01)	28547.67	(10852.58,65446.69)
Iraq	279.51	(60.52,785.80)	587.94	(129.09,1637.58)	5071.69	(1978.01,11925.28)	11664.41	(4604.11,27557.22)
Jordan	43.68	(9.92,122.81)	168.53	(38.63,467.70)	857.91	(346.20,2044.08)	3525.02	(1405.98,8059.78)
Kuwait	14.55	(3.37,39.77)	72.98	(16.86,197.04)	306.88	(117.29,695.57)	1294.81	(515.00,2925.28)
Lebanon	81.89	(18.56,229.83)	326.58	(71.07,891.74)	1660.35	(649.18,3961.60)	5793.53	(2308.50,13326.60)
Oman	7.94	(1.68,22.59)	14.10	(2.96,39.31)	187.53	(66.98,461.95)	344.69	(126.75,811.69)
Palestine	27.53	(6.34,77.08)	49.01	(10.77,140.90)	502.11	(188.32,1204.09)	1016.18	(398.26,2480.58)
Qatar	0.98	(0.20,2.85)	7.71	(1.55,22.51)	27.74	(9.92,68.63)	246.09	(88.66,610.66)
Saudi Arabia	79.63	(15.51,231.46)	176.47	(36.11,512.85)	1723.17	(604.43,4176.49)	4409.46	(1608.42,10677.50)
Syrian Arab Republic	188.70	(42.18,537.06)	288.46	(59.74,826.15)	3372.21	(1349.24,7867.66)	5949.21	(2299.30,13923.55)
Turkey	1271.11	(279.55,3510.44)	2708.70	(577.61,7473.56)	25047.37	(9741.73,58598.77)	52705.29	(20421.26,121531.48)
United Arab Emirates	3.58	(0.71,10.41)	25.55	(5.01,79.64)	99.06	(32.87,257.74)	823.69	(276.71,2174.83)
Yemen	89.19	(17.58,266.44)	307.23	(64.75,883.82)	2125.81	(789.01,5212.56)	6470.07	(2486.99,15532.87)
**Central Europe**								
Albania	67.95	(14.91,191.58)	195.32	(44.74,545.95)	1240.52	(483.44,2923.05)	3450.07	(1353.40,7938.47)
Bosnia and Herzegovina	100.94	(23.14,286.29)	252.69	(56.71,725.16)	2159.11	(856.08,4916.67)	4884.27	(1958.65,11313.51)
Bulgaria	293.37	(60.91,855.65)	389.92	(78.66,1150.84)	7119.89	(2823.78,16781.78)	8293.73	(3207.47,19169.06)
Croatia	256.57	(57.53,728.52)	382.95	(85.67,1127.90)	5160.79	(2065.43,11828.68)	6871.47	(2641.26,16008.55)
Czechia	474.47	(99.11,1335.13)	810.70	(179.88,2337.78)	9521.82	(3661.99,22054.41)	15177.02	(5971.20,34963.32)
Hungary	431.58	(88.45,1242.98)	579.23	(123.15,1714.80)	9304.69	(3712.34,21768.60)	11604.10	(4583.92,27508.88)
Montenegro	22.87	(4.91,63.34)	42.65	(9.39,121.60)	462.29	(186.06,1061.70)	880.32	(359.37,2009.76)
North Macedonia	53.41	(11.38,151.74)	94.49	(19.95,277.67)	1132.91	(446.77,2618.74)	2059.07	(807.01,4966.02)
Poland	1632.32	(359.17,4548.40)	2578.40	(582.40,7423.30)	32568.76	(12601.78,75292.99)	48055.08	(18580.53,113490.05)
Romania	542.28	(114.10,1500.81)	850.03	(189.26,2397.35)	12326.35	(4735.13,27455.33)	16874.96	(6513.79,39460.75)
Serbia	284.65	(59.81,810.80)	526.02	(111.30,1598.19)	5918.03	(2351.05,13579.78)	10984.09	(4341.15,25563.79)
Slovakia	147.24	(30.37,438.33)	198.90	(43.47,579.88)	3025.85	(1158.26,7342.97)	3977.60	(1517.03,9392.80)
Slovenia	74.08	(15.63,218.84)	167.93	(37.32,466.41)	1563.41	(612.17,3629.53)	3142.66	(1236.62,7048.14)
**Eastern Europe**								
Belarus	272.25	(61.23,768.71)	356.99	(75.99,1030.40)	5259.20	(2160.58,11769.47)	6778.09	(2778.66,15637.76)
Estonia	40.45	(8.53,115.68)	89.24	(19.97,258.41)	879.51	(336.85,2015.50)	1667.23	(650.74,3812.89)
Latvia	68.39	(14.80,200.38)	85.22	(19.89,249.60)	1446.09	(582.94,3263.86)	1687.55	(663.55,3955.13)
Lithuania	97.48	(21.29,274.43)	128.76	(28.42,358.68)	1871.63	(727.75,4248.53)	2370.56	(924.05,5461.58)
Republic of Moldova	59.04	(12.10,167.64)	121.31	(27.50,340.08)	1312.74	(523.28,3050.88)	2351.40	(952.52,5434.47)
Russian Federation	1836.14	(382.93,5229.99)	5072.22	(1170.27,14125.24)	42198.23	(16643.19,98760.26)	99050.60	(40332.03,218867.88)
Ukraine	1096.18	(235.06,3136.49)	1634.39	(359.34,4692.94)	22920.00	(9438.61,53040.43)	31088.40	(12488.87,73127.79)
**Western Europe**								
Cyprus	21.03	(4.52,60.58)	56.17	(12.48,158.28)	468.08	(179.22,1128.63)	1149.10	(465.26,2610.73)
Greece	546.54	(120.46,1529.68)	1316.68	(295.88,3719.36)	10739.44	(4461.64,24271.94)	20413.07	(8182.17,47128.04)
Israel	157.19	(34.51,449.60)	366.72	(83.38,1028.48)	3083.02	(1282.34,7169.91)	6117.65	(2414.82,14048.86)

Note:DALYs, disability-adjusted life years; SADD, Alzheimer's disease and other dementias attributable to smoking; “B&R”, the Belt and Road Initiative.

We found geographical differences in mortality and DALYs of SADD in the BRI countries. The mortality and DALYs of SADD were the highest for China, India, and Russian Federation in 1990 or 2019; the DALYs due to SADD in these three countries was 1.54 million (accounting for 38.78% of the global value) in 2019, which is 1.06 million more than that in 1990 (0.48 million, accounting for 24.70% of the global value). This increase accounted for 52.37% of the global DALYs growth value from 1990 to 2019. The country with the lowest mortality and DALYs in 2019 was Brunei Darussalam, a high-income country in Asia Pacific (3.38, 95% UI: 0.71 to 9.99 and 80.50, 95% UI: 28.96 to 196.77). The country with the largest increase in the number of DALYs due to SADD from 1990 to 2019 was China (828922.76, from 357257.80 to 1186180.56) and the largest increase in the proportion of DALYs due to SADD was seen in Qatar (787.13%, from 27.74 to 246.09). The only country with a decrease in the value of DALY due to SADD was Tajikistan (−20.64%, from 1366.52 to 1084.44).

**Fig. 1:**
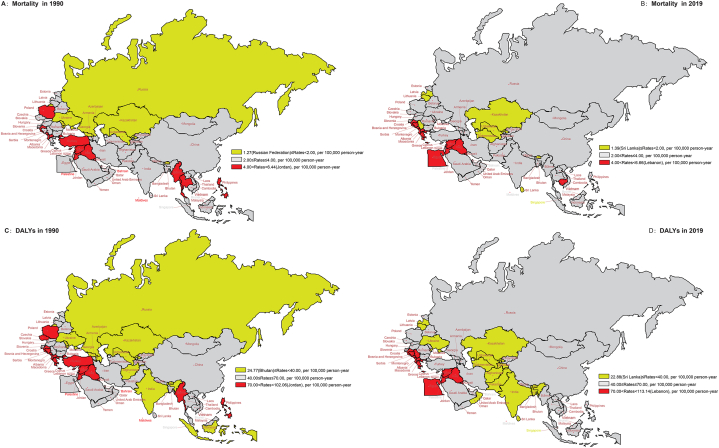
The ASRs of mortality and DALYs attributable to SADD for the BRI countries in 1990 and 2019 *(A) ASMR in 1990 (B) ASMR in 2019 (C)* ASDALR *in 1990 (D)* ASDALR *in 2019* Abbreviations: DALYs, disability-adjusted life years; SADD, Alzheimer's disease and other dementias attributable to smoking; ASRs, age-standardized rates; ASMR, age-standardized mortality rate; BRI, the Belt and Road Initiative.

The ASDALR of SADD showed a decrease from 1990 to 2019 both globally and in different SDI regions (Fig. 1). In 1990, the regions with high ASR mortality and DALYs were concentrated in North Africa and Middle East, Southeast Asia, Western Europe, and Central Europe, with Jordan having the highest ASR (6.44 per 100,000 and 102.06 per 100,000, respectively), Russian Federation having the lowest ASMR (1.27 per 100,000) and Bhutan having the lowest ASDALR (24.77 per 100,000). However, in 2019, the country with the lowest ASR mortality and DALYs were noted in was Sri Lanka (1.39 per 100,000 and 22.88 per 100,000, respectively), whereas the country with the highest values of ASR mortality and DALY was Lebanon (6.66 per 100,000 and 113.14 per 100,000, respectively).In 2019, ASDALR was the highest in Lebanon, Montenegro, and Bosnia and Herzegovina and the lowest in Uzbekistan, Bhutan, and Sri Lanka.

An increase in ASDALR from 1990 to 2019 was seen in 43.94% (29/66) countries, especially in the high-middle-SDI region, whereas a decrease was seen in 56.06% (37/66) countries, mainly comprising low-, low-middle, middle-, high-SDI regions. However, the ASDALR in Egypt, a middle-SDI region, showed an increase, with a maximum variation rate of 20.33 per 100,000, while the ASDALR in Burma, a low-middle-SDI region, showed a decrease, with a maximum variation rate of 37.86 per 100,000. Forty-one BRI members showed a general decrease in the burden of SADD from 1990 to 2019 (all *P* < 0.05). However, the ASDALR of SADD in India showed a decline from 1990 to 2019 (from 32.84/105 to 29.35/105), but increased from 2010 to 2019 (from 28.29/105 to 29.35/105). On the other hand, the ASDALR of SADD in China increased from 1990 to 2019 (from 55.50/105 to 66.18/105), but decreased from 2010 to 2019 (from 68.97/105 to 66.18/105). See Supplementary Table S2 for more details.

**Fig. 2:**
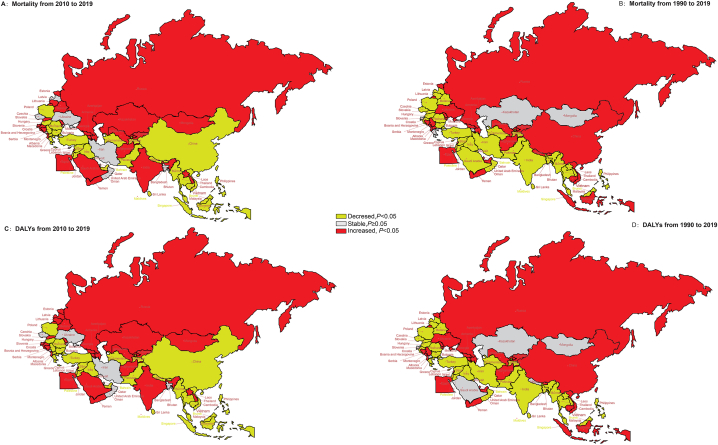
The temporal trend in the ASRs of mortality and DALYs of SADD for 1990–2019 and 2010–2019 in the BRI countries (A) The AAPC of ASMR from 1990 to 2019 (B) The AAPC of ASMR from 2010 to 2019 (C) The AAPC of ASDALR from 1990 to 2019 (D) The AAPC of ASDALR from 2010 to 2019 Abbreviations: DALYs, disability-adjusted life years; SADD, Alzheimer's disease and other dementias attributable to smoking; AAPC, average annual percent change; ASRs, age-standardized rates; ASMR, age-standardized mortality rate; ASDALR, age-standardized disability-adjusted life years rate; BRI, the Belt and Road Initiative.

The temporal trend of ASR of mortality and DALYs due to SADD for 1990–2019 and 2010–2019 in BRI countries is shown in Fig. 2. From 1990 to 2019, the fastest decline in the ASMR (AAPC: 2.89%; 95% CI: 2.98% to −2.80%) and DALYs (AAPC: 2.6%; 95% CI: 2.79% to −2.59%) was seen in Sri Lanka, Southeast Asia. In contrast, the fastest increase in ASMR (AAPC: 2.24%) and DALYs (AAPC: 1.97%) was seen in Russian Federation, Eastern Europe (all *P* < 0.001). From 2010 to 2019, the fastest decline in ASDALR was seen in Sri Lanka (AAPC: 2.80%; 95% CI: 3.11% to −2.48%) and Burma (AAPC: 2.80%; 95% CI: 2.88% to −2.72%), both in Southeast Asia. Furthermore, the fastest increase in ASDALR was seen in Kyrgyzstan, Central Asia (AAPC: 3.17%; 95% CI: 2.74 to 3.60; *P* < 0.001). We noted a substantial downward trend in ASMR and ASDALR due to SADD during 1990–2019 as well as the final decade (2010–2019) both globally and in different SDI regions (all *P* < 0.05, except for ASDALR and ASMR from 1990 to 2019 in high-middle-SDI region). However, a decrease in ASMR from 1990 to 2019 was noted only in two out of nine countries in Central Asia and in none of the countries in Eastern Europe (all *P* < 0.05). See Supplementary Table S3 for more details.

**Fig. 3:**
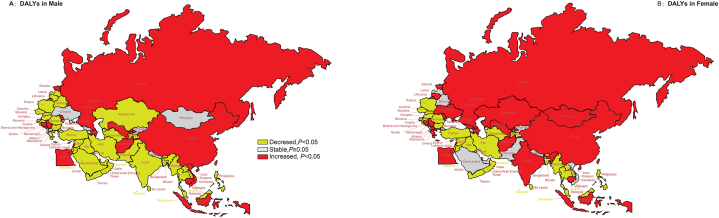
The temporal trend in the ASDALR of SADD, stratified by gender for 1990–2019 in the BRI countries (A) The AAPC of ASDALR in male (B) The AAPC of ASDALR in female Abbreviations: DALYs, disability-adjusted life years; SADD, Alzheimer's disease and other dementias attributable to smoking; AAPC, average annual percent change; ASDALR, age-standardized disability-adjusted life years rate; BRI, the Belt and Road Initiative.

Fig. 3 shows the AAPC of ASDALR due to SADD in males and females in each BRI country for 1990 to 2019. The ASDALR in males decreased markedly globally and in different SDI regions, including most members from South Asia (4/5), Southeast Asia (7/10) and high-SDI countries in Asia Pacific (2/2), North Africa and Middle East (13/16), Central Europe (9/13), and Western Europe (3/3). A marked increase in ASDALR was noted in BRI countries of East Asia (1/1), Central Asia (2/9), Southeast Asia (3/10), North Africa and Middle East (3/16), Central Europe (3/13), and Eastern Europe (3/7) (all *P* < 0.01).

The ASDALR in females showed a marked decrease both globally and in certain low-middle-SDI and high-SDI regions, including most BRI countries in South Asia (3/5), Southeast Asia (7/10), and high-SDI countries of Asia Pacific (2/2), North Africa and Middle East (11/16), Central Europe (7/13), and Western Europe (2/3). On the other hand a marked increase in ASDALR in females was noted in low-SDI and high-middle-SDI regions, including most BRI countries in East Asia (1/1), Central Asia (5/9), and Eastern Europe (5/7) (all *P* < 0.01).

The AAPC in ASDALR among females was greater than that among males in 20 countries, but lower than that among males in 19 countries (all *P* < 0.05). (Supplementary Table S4).

**Fig. 4:**
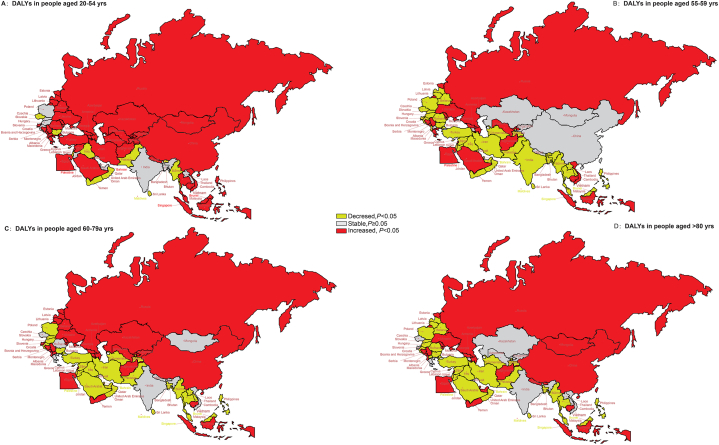
The temporal trend in the DALYs rate of SADD, stratified by age for 1990–2019 in the BRI countries *(A) The AAPC of DALYs rate in people aged 20–54 years (B) The AAPC of DALYs rate in people aged 55–59 years (C) The AAPC of DALYs rate in people aged 60–79 years (D) The AAPC of DALYs rate in people aged ≥80 years* Abbreviations: DALYs, disability-adjusted life years; SADD, Alzheimer's disease and other dementias attributable to smoking; AAPC, average annual percent change; BRI, the Belt and Road Initiative.

Fig. 4 shows the long-term trends in age-stratified DALYs due to SADD in BRI countries for the period 1990–2019. A downward trend of DALYs was noted in all age groups of 11 BRI countries that are classified as low-SDI and high-SDI regions: Pakistan in South Asia; Maldives, Burma, Philippines, and Sri Lanka in Southeast Asia; Iraq, Oman, and Qatar in North Africa and Middle East; Bulgaria in Central Europe; and Cyprus and Israel in Western Europe. On the other hand, an increasing trend in the DALYs was noted for all age groups in the 14 member countries: Azerbaijan and Georgia in Central Asia; Cambodia and Indonesia in Southeast Asia; Afghanistan, Egypt, and Lebanon in North Africa and Middle East; Bosnia and Herzegovina, Montenegro, and Serbia in Central Europe; Estonia, Republic of Moldova, Russian Federation, and Ukraine in Eastern Europe (all *P* < 0.01).

For the age group of 20–54 years, a decreasing trend was noted in the DALYs due to SADD in 13 countries classified as low-SDI regions (all *P* < 0.05). For people aged 60 years and above, a decrease in DALYs was noted globally and in different SDI regions, except for high-middle-SDI regions, including 18 BRI countries, including China and Russian Federation (all *P* < 0.05). See Supplementary Table S5 for more details.

## 4 Discussion

Smoking is a dangerous habit, which not only has negative effects on the health of smokers themselves but also imposes a huge burden of disease on the society [23]. WHO reports that smoking-related diseases cause millions of deaths every year and that besides being the cause of premature deaths, smoking imposes economic burdens on both families and societies [24]. The treatment of diseases caused by smoking involves utilization of a large amount of medical resources and expenses, which is borne by individuals as well as shared by society, and an increase in these expenses puts pressure on the healthcare system, affecting socio-economic development [23,25].

Dementia is a serious neurodegenerative disease characterized by the loss of cognitive abilities and a decline in daily life skills, and smoking has been shown to have a significant impact on the health of dementia patients [26]. Smoking accelerates the progression of dementia and worsens its severity [27]. Chemicals in tobacco products have toxic effects on the brain, damaging memory, thinking, and cognitive abilities. Smoking also increases the risk of other health issues such as cardiovascular and respiratory diseases, thus further compromising the overall health of dementia patients [28,29].

The pathogenesis of dementia due to smoking involves multiple factors, such as atherosclerosis, inflammation, and oxidative stress. First, smoking causes an increase in blood cholesterol levels and impairs vascular function, leading to atherosclerosis and the formation of blood clots; these changes lead to a reduction in the blood supply to the brain and an increased risk of dementia [30]. Second, certain harmful compounds in cigarettes trigger inflammatory reactions by the immune system, and long-term smoking can lead to chronic inflammation, which increases the risk of neuronal damage and death, accelerating the progression of dementia [31]. Third, some harmful substances in cigarettes compromise the ability of cells to cope with oxidative stress, leading to the accumulation of oxygen free radicals within the cells, which, in turn, causes oxidative damage to DNA and proteins, promotes damage to neurons and brain cells, accelerates the process of neurodegeneration, and promotes the occurrence of dementia [32]. Fourth, smoking causes damage to the blood-brain barrier, allowing harmful substances and toxins to enter the brain, interfere with normal neuronal function, and disrupt neural transmission, thus leading to dementia [33,34]. Recent studies have also shown that smoking interferes with the normal synthesis and release of neurotransmitters, disrupting the balance of neural transmission, thereby affecting cognitive function and memory and increasing the risk of dementia [35].

The harmful effects of tobacco on human health have been widely recognized, and tobacco control policies have been implemented worldwide. As members of BRI, more and more countries have started realizing the importance of tobacco control policies in health management. In China, smoking behavior is restricted through laws such as the "People's Republic of China Smoking Control Regulations," increased use of warning labels, launching of campaigns to raise public awareness regarding the dangers of smoking, strengthening the regulation of tobacco sales, and provision of smoking cessation services and support to help smokers quit [[36], [37], [38]]. Russia's tobacco control policies include banning smoking in public places, use of warning labels on tobacco packaging, implementing tobacco taxes to increase the cost of tobacco products, and supporting smokers in quitting through education and smoking cessation services [39]. India, one of the world's largest consumers of tobacco products, employs various measures in its tobacco control policies, including laws, taxes, public education, and propaganda [40].

The results of this study showed that in the BRI countries, the disease burden was positively correlated with the population size. Both in 1990 and 2019, China, Russia, and India—the three populous countries—consistently had the highest disease burden and increases, and their contribution to GBD in 2019 was greater than that in 1990, with this increase accounting for more than half the global increase. The increase in dementia cases can be attributed to the aging of the population and possibly to the challenges to local tobacco control policies [41]. Additionally, the influence and interests of the tobacco industry also make it difficult to promote and effectively implement tobacco control policies [42]. Often, there is a confrontation between the conflicting interests of tobacco companies and the government, creating pressure and resistance for the government in formulating and implementing tobacco control policies [43]. This is compounded by the substantial contribution made by the tobacco industry to the national economy, because of which some countries may be concerned about the negative impact of tobacco control policies on economic development, leading them to adopt a reserved attitude towards tobacco control measures [44]. For example, in China, significant challenges to smoking control are the large size and profitable of the tobacco industry, the prevalence of smoking culture in some regions, and the massive population [37,45]. Similarly, in India, although some tobacco control measures have achieved positive results to some extent, smoking remains widespread and the Indian government still needs to intensify its efforts to implement stricter tobacco control policies and measures to further improve public health [46].

The results of this study indicate an increase in the DALYs and ASDALR of SADD in 29 BRI countries, including China and Russia, a finding that is inconsistent with the trend of “rate decrease and quantity increase” observed worldwide and in different SDI regions. This indicates that the implementation and effectiveness of tobacco control policies in some BRI countries still remain inadequate [47]. Although some countries have taken a series of measures, due to the uneven rate of development within a country, awareness of the dangers of smoking may be inadequate among people residing in remote, underdeveloped areas, and in such areas, the enforcement of tobacco control policies may not be strict enough; this leads to high smoking rates and limited effectiveness of tobacco control policies in certain regions [48].

Our findings indicated sex-related differences in the burden and trends of SADD in BRI countries. Previous studies have shown that male dementia patients are more likely to continue smoking than their female counterparts due to "tobacco dependence" and higher social and environmental pressures and that female dementia patients are more likely to choose smoking cessation on realizing the health hazards of smoking [49]. Our results show that unlike the global trend of decreasing ADD burden for both genders with a higher annual decrease rate in females, 20 countries, including China, Russia, and India, had a higher annual growth of ASDALR among females as compared to males, whereas 19 countries, including Turkey, United Arab Emirates, and Poland, had a lower annual decrease ASDALR of females as compared to males. These findings can be attributed to the worrisome trend of female smokers worldwide gradually surpassing males in the risk of dementia as well as the changes in the smoking culture in these countries. Specifically in India, the changes in Indian society and the improvement of women's status has been paralleled by a rise in the smoking rate among women, making it one of the countries with the highest number of female smokers in the world, further exacerbating the growth of SADD burden [40]. These results suggest that among BRI members, countries such as China, Russia, India, and Turkey should play a more proactive role in dementia prevention and control and highlight the need for joint research undertakings to identify solutions and develop strategies for more effective prevention and intervention [44]. In addition, the WHO and BRI countries should increase support for these countries, including providing funding and technical support, to promote the implementation of dementia prevention and control measures [43].

Our findings also revealed age-related differences in the burden of SADD in BRI countries. The burden of dementia diseases has been previously shown to increase with age: prevalence of dementia in China among people over 60 years old is about 2.5% and has been reported to increase exponentially with age [50,51]. The increased prevalence of dementia among elderly individuals may be related to the biological and metabolic changes during the aging process as well as the higher likelihood of comorbidities such as hypertension, diabetes, and cardiovascular and cerebrovascular diseases, which may increase the risk of developing ADD [51,52]. Our study found that unlike the decreasing trend of SADD burden in the population aged 60 years and above seen globally, SADD DALY rates in BRI countries, including China and Russia, are on the rise. This is mainly due to the aging of the population in these countries over the past 30 years. In recent years, the population aged 60 years and above in China has increased from less than 7% to over 20%, and since the burden of dementia is mainly concentrated in the elderly population aged 60 years and above, this has led to an explosive growth in the burden of dementia diseases [53]. Apart from this, the prevalence of dementia among young people aged 20–54 years has also increased, indicating the need for different prevention and treatment strategies for different age groups in order to effectively address this global health challenge and provide better support and care for individuals and society [1].

The strength of this study lies in the analysis of the burden of smoking-induced dementia among residents of different ages and genders in BRI countries from the perspective of nation-level efforts in countries with long-standing political and economic ties. This study focuses on the burden of smoking-induced dementia through the horizontal strong connections of trade, as well as the vertical descent of experience shared among countries with different SDI levels and the differences between countries and their corresponding SDI levels. Our study also explores tobacco control policies for preventing dementia in the context of deepening regional cooperation and high-quality development of trade, thus promoting the initiative of the shared future of the community in the health field [37].

There are a few limitations to this study. This study is based on a secondary analysis of GBD2019 data, which have inherent limitations that cannot be eliminated. The GBD database lacks details on the pathological staging and classification of SADD. Hence these aspects could not be included in the present study. In the future, BRI countries can use economic development as an impetus to drive the construction of information-based disease-monitoring systems, providing sufficient support for the estimation of disease burden and policy adjustments.

In brief, a quantitative analysis of the diversities of SADD burden and an understanding of the trends in its geographic, sex, and age distribution in BRI countries may facilitate the development of strategies for the prioritization of dementia prevention and burden reduction. This in turn would help international organizations work towards achieving the sustainable development goals of improving health and well-being for all. Through comprehensive and collaborative efforts, it is possible to reduce the burden of dementia and improve the quality of life for millions of individuals and their families.

## Ethics declarations

Review and/or approval by an ethics committee was not needed for this study because animal experiments or clinical studies are not required for this study.

## Data Availability statement

To download the data used in our analyses, visit the 2019 Global Health Data Exchange website https://vizhub.healthdata.org/gbd-results/

## CRediT authorship contribution statement

**Zheng Luo:** Writing – original draft, Funding acquisition, Formal analysis. **Xin He:** Writing – original draft, Validation, Data curation. **Huihui Lv:** Validation, Funding acquisition, Data curation. **Qizhe Wang:** Software, Data curation. **Wenchang Jia:** Visualization, Software. **Yajun Zhao:** Visualization, Software, Methodology. **Xinyi Li:** Visualization. **Jiali Yu:** Visualization, Data curation. **Hongyu Hao:** Visualization, Software. **Yun Bao:** Data curation. **Nuo Chen:** Data curation. **Xiaopan Li:** Writing – review & editing, Validation, Supervision, Methodology, Conceptualization.

## Declaration of competing interest

The authors declare that they have no known competing financial interests or personal relationships that could have appeared to influence the work reported in this paper.
